# Interactive decision support for esophageal adenocarcinoma screening and surveillance

**DOI:** 10.1186/s12876-019-1022-0

**Published:** 2019-06-27

**Authors:** Thomas L. Vaughan, Lynn Onstad, James Y. Dai

**Affiliations:** 1 Division of Public Health Sciences, Fred Hutchinson Cancer Research Center, Seattle, WA 98109 USA; 2 Program in Cancer Epidemiology, M4-B874, 1100 Fairview Ave N, Seattle, WA 98109 USA

**Keywords:** Risk calculator, Risk prediction, Esophageal cancer, Esophageal adenocarcinoma, Barrett’s esophagus, Absolute risk, R language, Shiny application, Web application

## Abstract

**Background:**

A key barrier to controlling esophageal adenocarcinoma (EAC) is identifying those most likely to benefit from screening and surveillance. We aimed to develop an online educational tool, termed IC-RISC™, for providers and patients to estimate more precisely their absolute risk of developing EAC, interpret this estimate in the context of risk of dying from other causes, and aid in decision-making.

**Results:**

U.S. incidence and mortality data and published relative risk estimates from observational studies and clinical trials were used to calculate absolute risk of EAC over 10 years adjusting for competing risks. These input parameters varied depending on presence of the key precursor, Barrett’s esophagus. The open source application works across common devices to gather risk factor data and graphically illustrate estimated risk on a single page. Changes to input data are immediately reflected in the colored graphs. We used the calculator to compare the risk distribution between EAC cases and controls from six population-based studies to gain insight into the discrimination metrics of current practice guidelines for screening, observing that current guidelines sacrifice a significant amount of specificity to identify 78–86% of eventual cases in the US population.

**Conclusions:**

This educational tool provides a simple and rapid means to graphically communicate risk of EAC in the context of other health risks, facilitates “what-if” scenarios regarding potential preventative actions, and can inform discussions regarding screening, surveillance and treatment options. Its generic architecture lends itself to being easily extended to other cancers with distinct pathways and/or intermediate stages, such as hepatocellular cancer. IC-RISC™ extends current qualitative clinical practice guidelines into a quantitative assessment, which brings the possibility of preventative actions being offered to persons not currently targeted for screening and, conversely, reducing unnecessary procedures in those at low risk. Prospective validation and application to existing well-characterized cohort studies are needed.

**Electronic supplementary material:**

The online version of this article (10.1186/s12876-019-1022-0) contains supplementary material, which is available to authorized users.

## Background

Incidence of esophageal adenocarcinoma (EAC) has risen markedly in many western countries. Most cases can be attributed to known risk factors, such as symptomatic gastroesophageal reflux (sGERD), central obesity, cigarette smoking and family history [1]. Nevertheless, the relative rarity of the cancer, combined with the cost and invasiveness of upper endoscopy for identifying early cancers and high-risk pre-cancers (e.g., Barrett’s esophagus (BE) with dysplasia or genomic abnormalities) [2, 3] make it challenging to define effective screening and surveillance strategies [4].

A key barrier has been identifying those most likely to benefit from endoscopy or newer non-endoscopic tissue sampling methods [5, 6]. Clinical practice guidelines vary by country and professional society regarding criteria for initial screening for BE or EAC, as well as the definition of BE [7, 8]. Furthermore, none consider the strong effect of age on EAC incidence except in defining a fixed age threshold, and all tend to treat the remaining risk factors as equally important. For example, 2016 American College of Gastroenterology (ACG) guidelines [7] suggest that screening may be considered among men with sGERD plus two or more other specified risk factors for BE or EAC. However, this qualitative approach excludes the approximately 47% of all EAC that present in persons without significant sGERD, who may be at increased risk due to other factors, and does not take advantage of known quantitative relationships (i.e., strength of association and dose-response) between EAC incidence and sGERD, smoking and obesity, for example [9].

To address this barrier, an online Interactive and Contextual Risk Calculator (IC-RISC™; https://ic-risc.esocan.org) was developed to take advantage of existing knowledge from observational studies and clinical trials to estimate more precisely an individual’s absolute risk of developing EAC over a ten-year period, and to convey this estimate in the context of risk of dying from other cancers or from common causes such as injury, stroke or heart disease. Using this calculator, we compared the risk distribution between EAC cases and population-based controls from six studies in the Barrett’s and Esophageal Adenocarcinoma Consortium (BEACON; https://beacon.esocan.org) to contrast discrimination metrics of current guidelines vs. more stringent thresholds that might be used.

### Implementation

Information required to calculate absolute risk of EAC, adjusting for competing mortality, includes i) incidence and mortality rates of EAC; ii) all-cause mortality rates; and iii) relative risks (RR), 95% confidence intervals (CI) and prevalence for each risk factor. These data are input from three spreadsheet files. Given the wide variation in risk of EAC by demographic factors, the population incidence and mortality rates are age-, sex- and race-specific, rather than being model-based. Similarly, since EAC incidence is substantially higher among persons with BE, and the constellation and strength of risk factors that predict EAC are quite different from the general population, input parameters are stratified by diagnosed BE status (Additional file 1: Figure S1 and Tables S1 and S2).

**Fig. 1:**
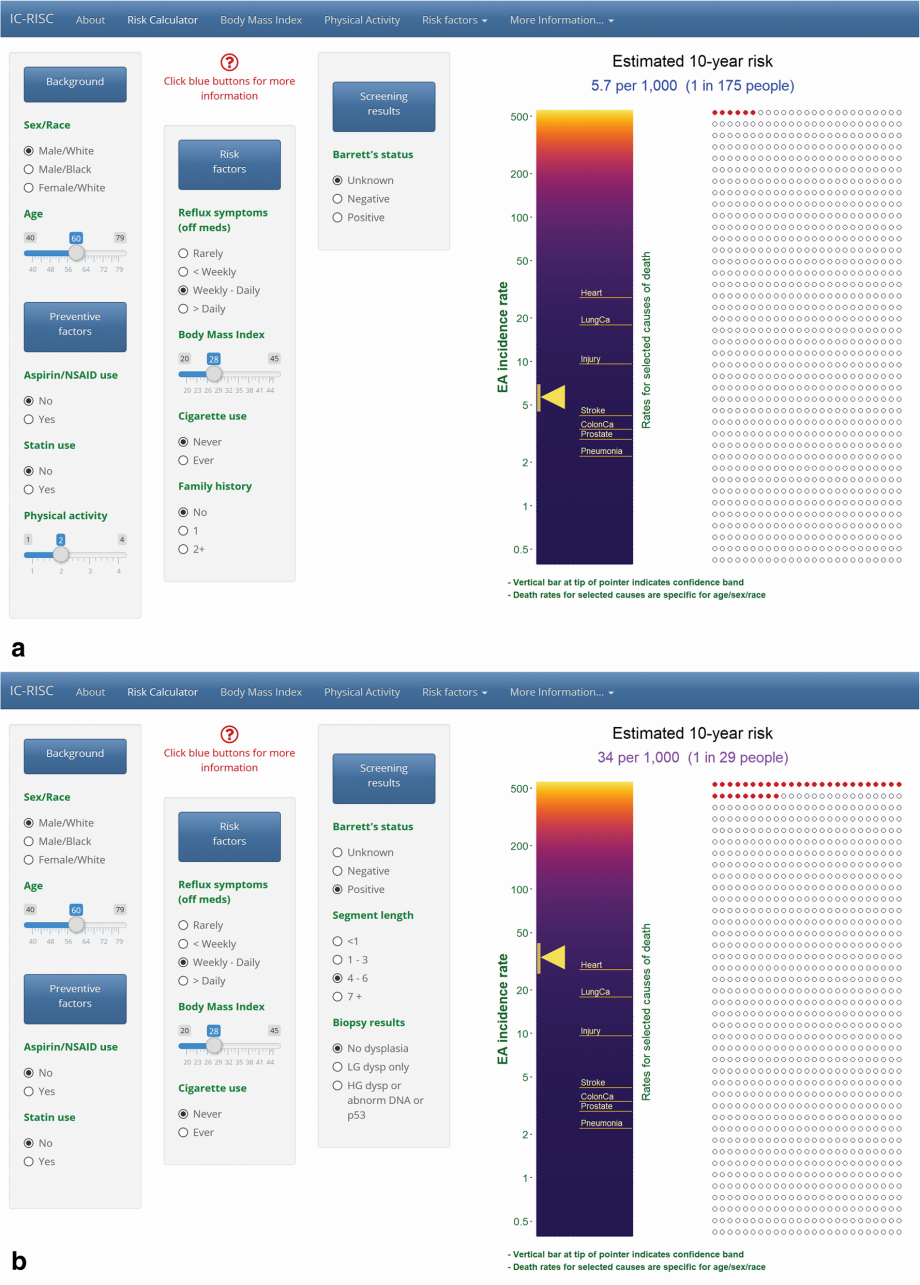
Risk calculator tab. In example **a**) A 60-year-old white male with moderate physical activity, non-use of NSAIDs or statins, no family history of BE or EAC, weekly – daily reflux symptoms, a body mass index (BMI) of 28 (“overweight”), who never smoked cigarettes, and has not been screened (BE status unknown) is estimated to have a 10-year risk of developing EAC of 5.7 per 1000, or 1 in 175 people of similar characteristics. This is higher than the 10-year risk of dying from colon cancer and stroke for a 60-year-old white male, but lower than that from injury or heart disease. In example **b**) this same individual has undergone an upper endoscopy and found to have a visible Barrett’s segment length of 5 cm, but no evidence of dysplasia. His 10-year risk is now estimated to be 34.0 per 1000 (1 in 29 people) which is approximately equal to his risk of dying from heart disease

#### Incidence and mortality

The mean incidence and mortality rates by sex and race (white, black) in 5-year age groups (ages 40–84, and 85+) were obtained from the SEER database of 18 cancer registries for the years 2010–2015 [10]. Overall and cause-specific mortality rates by sex and race in 5-year age groups for the year 2014 were obtained from National Center for Health Statistics [11]. Estimated annual EAC incidence among persons with BE varies greatly depending on cohort definition and study design, but recent reports range from 0.19–0.41% among men and women [12–14]. In our calculations we used an incidence of 0.31% per year for white men (including those with dysplasia), reported from a large (*n* = 8, 929) population-based cohort of persons with BE [15]. We applied this figure to the 60–64 year age group of white males, a typical mean age of diagnosis [15–17]. Incidence was assumed to vary from this figure by a factor of 1.04 per year of age [18, 19]. EAC incidence among white women with BE was estimated to be 0.4 times the rate of white men [15]. There is little information on black males with BE; assuming that a portion of their approximately four-fold overall lower risk of EAC can be attributed to lower risk at the BE stage, we estimated their EAC incidence at 0.75 times that of white males. For ease of use in calculations, incidence and mortality rates were modeled using a third-degree polynomial (Additional file 1: Figure S2).

#### Risk factors

Risk factors for EAC were identified from the literature. In the general population, sGERD, obesity, cigarette smoking, and family history of BE or EAC were the major factors associated with increased risk (Additional file 1: Table S1) [20–23]. Conversely, physical activity, use of non-steroidal anti-inflammatory drugs (NSAIDs) and statin drugs have been associated with decreased risk in observational studies and randomized clinical trials [5, 24–31]. Estimates of strength of association (e.g., RR), 95% CI, and prevalence were taken in most instances from large meta-analyses of pooled individual data from population-based case-control studies (noted by bold type in Additional file 1: Tables S1 and S2).

In the setting of BE, clinical (Barrett’s segment length), histopathologic (high-grade and confirmed low-grade dysplasia) and molecular/genomic abnormalities were the dominant predictors of progression (Additional file 1: Table S2) [3, 12, 14, 16–19, 26, 32–41] with smoking and body mass index (BMI) playing more minor roles [18, 19, 33, 38]. In contrast, NSAIDs and statins exhibited inverse associations with EAC similar to the general population, based on a clinical trial and a cohort study for NSAIDs [29, 34] and a meta-analysis of 11 studies for statins [24]. Little is known regarding physical activity and family history and progression risk in BE, so these factors were omitted. Regarding sGERD, the RR used in the calculator for progression to EAC is estimated as one half of the trend coefficient for the general population. (For additional details see Additional file 1).

#### Estimation of absolute risk

Absolute risk of EAC over 10 years, adjusting for competing risk of death, was calculated according to methods described by Hsu, et al., and others [42–44]. First, the baseline hazard rate, defined as the hazard rate for individuals whose risk factors are at the lowest risk level, was calculated from the age-, sex- and race-specific EAC incidence rates and the population attributable risk calculated using the risk factors and prevalence from Additional file 1: Table S1. Second, the relative risk for an individual was calculated as the product of the relative risks attributed to each risk factor. Finally, the baseline hazard rate, relative risk, and risk of death from competing causes were combined over 10 years to yield adjusted absolute risk of EAC.

95% CIs were calculated from standard errors using the delta method, thus accounting for both direct effects of uncertainty in RR estimates, and effects on the population attributable fraction. IC-RISC™ is written in the R programming language [45] using the Shiny [46] platform for web interactivity. The application is compatible with most computers, tablets and mobile devices, and uses a color blind-friendly palette. It is available under an open source license for academic/non-profit use.

#### Distribution of 10-year risk in EAC cases and population controls

To estimate how well the calculator discriminates between persons who have developed EAC and controls from the general population, we used individual harmonized exposure data from six population-based studies from the BEACON consortium with reasonably complete data on the key risk factors (sGERD, BMI, cigarette use, and NSAID use). These included the US Multicenter Study (western Washington center) [47], FINBAR (Northern Ireland and Ireland) [48], Los Angeles County Multi-ethnic Study [49], Australian Cancer Study [1], the Study of Digestive Health (Queensland, Australia) [50], and the Study of Reflux Disease (western Washington) [51]. Together, these studies included 495 EAC cases and 1376 controls. The joint distribution of these factors was taken as fixed, since the studies were population-based. Multiple imputation was used to fill in missing data (missingness = 24 for BMI, 81 for frequency of heartburn and/or reflux, 0 for ever smoking and 5 for NSAIDs.) Data was not available from these studies regarding physical activity, use of statins or family history of BE or EAC. Levels of these factors among U.S. population controls were estimated from other sources [26, 52, 53] and randomly assigned to controls in accordance with these distributions. Corresponding levels among cases were randomly assigned in such a way that their distribution was consistent with the RR estimate for each risk factor and level. For example, given 33% of the population 40+ years old in the U.S. are estimated to use statins [28]; approximately 22% of the cases were randomly assigned to be users, yielding the predetermined odds ratio of 0.57 [24].

**Fig. 2:**
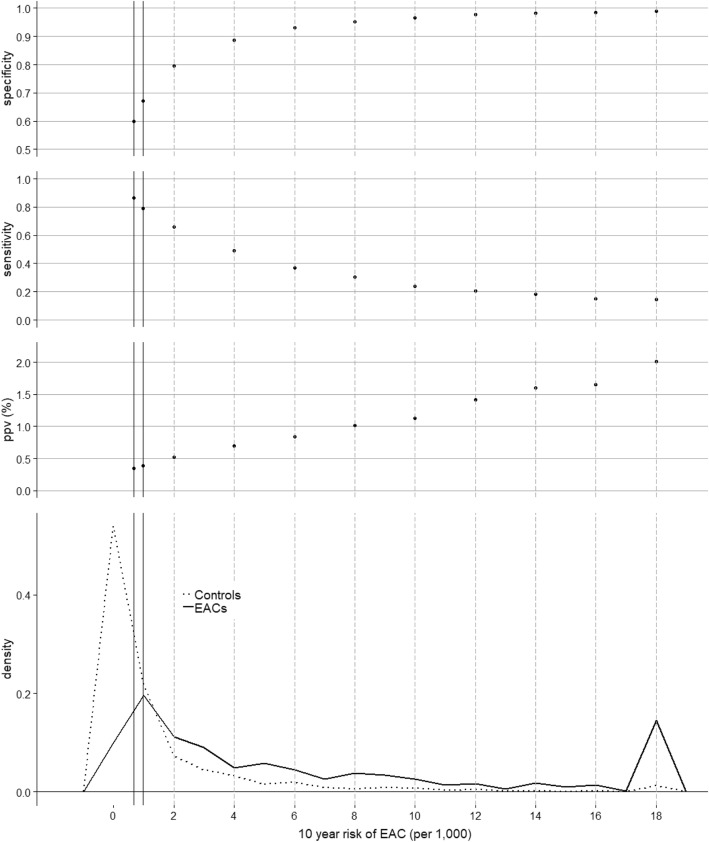
Metrics describing estimated 10-year risk in EAC cases and controls based on data from six population-based studies in the BEACON consortium. The bottom panel shows the distribution of 10-year risk estimates by case status. The solid vertical lines represent examples of an individual for whom current ACG guidelines suggest that screening endoscopy be considered. The first (10-year risk = 0.66/1000) is for a 40-year-old male with weekly-daily reflux and two additional risk factors (white and BMI > 25). The second (10-year risk = 0.97/1000) is for a 50-year-old male with weekly-daily reflux and with two different additional risk factors (age > 50 and positive cigarette smoking history). The top, second and third panels show how specificity, sensitivity and positive predictive value (ppv), respectively, vary according to possible thresholds for further action

Controls were ascertained in each study in such a way that their age and sex distribution was similar to that of the cases. Therefore white controls were re-weighted to represent the age and sex distribution of the white U.S. population from the SEER 18-registry population [10]. None of the studies included sufficient numbers of black participants to yield stable exposure distributions; for the purposes of Fig. 2, case and control records for black male participants were simulated to have the same risk factor distribution as white males of the same age, and weighted so that they were representative of the distribution of the black male EAC cases and population controls from the same SEER 18-registry area. Calculations were carried out in the R programming language.

## Results

The application interface consists of six tabs, including the calculator itself (Fig. 1.) Values for demographics, risk and preventive factors are entered on the left side of the page. Estimated 10-year probability of developing EAC is displayed on the right side in two ways: as a “thermometer” with color coded risk (on a log scale), along with an estimate of its uncertainty; and as a set of 1000 circles depicting the expected number who will (red fill) and will not (unfilled) develop the cancer in the next ten years. Any change in the predictors on the left is immediately reflected in the risk estimates on the right. The risk factors available for input and used in calculations depend on whether BE has been diagnosed. Figure 1 illustrates inputs and 10-year risk for an individual who has not been screened (BE status unknown) (Fig. 1a), and one who has been diagnosed with BE (Fig. 1b) (See legend for details.) Mortality rates for selected causes of death are displayed in the center of the thermometer; these are specific for age, sex and race (black/white), but do not consider non-demographic risk factors.

The landing page is the “About” tab describing the purpose and background. Tabs are available to calculate BMI and categorize usual physical activity, which are not usually known. The “Risk factors” tab includes three options for displaying the input data used: a graph (Additional file 1: Figure S1, of the RR estimates and 95% CIs for each factor, stratified by the presence of diagnosed BE (unknown/negative vs. positive); and detailed tables (Additional file 1: Tables S1 and S2, containing all the RR data with supporting references. Reliable risk factor data specific for black females and other demographic groups of either sex are not currently available. The “More information” tab contains an option for viewing the age-, race- and sex-specific incidence and mortality rates used in the calculator (stratified by BE status) and all-cause mortality (Additional file 1: Figure S2) Additional menu options include contacts, version history, and licensing information.

Figure 2 illustrates the distribution of 10-year risk estimates for EAC cases and controls using data from population-based BEACON studies, along with estimates of specificity, sensitivity and positive predictive value (ppv) according to increasingly more stringent thresholds for further action. The area under the associated ROC curve (not shown) is 0.81 (95% CI = 0.79–0.83.) When applied to the general population, as in Fig. 2, the calculator provides insights into the performance of current practice guidelines (two examples of which are represented by vertical solid lines, see legend) [7], which sacrifice a significant amount of specificity in order to capture 79–86% of eventual cases. A small increase in action threshold, for example to 2 per 1000, would potentially eliminate almost half of procedures or tests (reducing 40% false positives to 20%), while only reducing sensitivity to 66%; whereas an increase to 4 per 1000, would further reduce false positives to 12%, with a sensitivity of 49%. Unfortunately, given the rarity of EAC, only with higher thresholds of 8 per 100,000 or more does the ppv surpass 1%.

It is notable that absolute risks of greater than 2.0 are readily reached by persons not included in current guidelines. For example, a 65-year-old white female ex-smoker with frequent (“> daily”) reflux symptoms, no family history, and a BMI of 31 has an estimated risk of 4.4 per 1000. Similarly, a 60-year-old non-smoking white male without reflux symptoms (“rarely”), a BMI of 31 (obese category I) and one first degree relative with BE/EAC has an estimated risk of 4.8 per 1000. Finally, a 65-year-old ex-smoking black male with occasional reflux symptoms (“< weekly”), no family history, and a BMI of 36 (obese category II) has an estimated risk of 2.1 per 1000.

Given that almost half of EAC cases in the general population do not report significant symptoms of reflux [9], we investigated the performance of the risk calculator in the subset of the cases (46%) and controls (70%) from the BEACON studies who reported sGERD only occasionally (“< weekly”) or rarely (Additional file 1: Figure S3) The overall discriminatory ability in this subset was similar to that in the entire dataset, though slightly more modest, with an area under the ROC curve of 0.78 (95% CI = 0.75–0.81.) While a specificity of 95% was reached at a lower risk threshold than in the entire dataset (about 4 vs. 8 per 1000 over ten years), the sensitivity at the lower threshold in this low-sGERD subset was only half (25%) that of the entire dataset (50%), and the ppv did not rise above 0.5%.

## Discussion

This educational tool was developed to facilitate shared decision-making between a health provider and patient regarding: i) how personal risk of EAC fits into the “bigger picture” of health and disease, ii) whether preventive actions are indicated to possibly reduce risk of EAC and other conditions, and iii) whether additional tests and procedures might be warranted to identify and manage those with higher risk profiles [54].

A strength of IC-RISC™ is the robust data on which it is based. Incidence rates of EAC were based on the SEER 18 registries, which cover more than a quarter of the US population, and RR and prevalence estimates for the general population calculations came from large individual pooling efforts (including between 900 and 1500 cases). This contrasts with previous efforts in the general population which have both estimated associations and created risk models using relatively small individual studies, often with varying availability and comparability of potential predictors [42, 55, 56]. For example, in a prospective cohort study with over 350,000 participants, 220 developed EAC over up to eight years of follow up; BMI, smoking and a prior esophageal condition or treatment were found to be predictive and included in a risk score [56]. In another study of 189 EAC cases, final predictive variables included sGERD and/or use of anti-reflux medication, BMI, tobacco smoking, duration of living with a partner, previous diagnoses of esophagitis and diaphragmatic hernia, and previous surgery for esophagitis, diaphragmatic hernia or severe reflux or gastric or duodenal ulcer [55].

In persons with BE, the evidence base for predicting progression to EAC is more limited, and results often conflicting. Although many of the studies informing RR estimates are cohort studies, which have many strengths including internal consistency and lack of recall bias, the small number of outcomes for a rare disease such as EAC, varying designs, limited risk factor information and questions about generalizability remain important issues. For example, in a report on a non-HGD cohort with 154 EAC/HGD outcomes, three factors were observed to be significantly predictive: segment length, smoking and confirmed low-grade dysplasia [38]. Unexpectedly, this study also observed that progressors had significantly lower BMI (*p* = 0.012) and obesity (*p* = 0.049, RR = 0.68) than non-progressors, which is inconsistent with most observational studies [18, 55, 57]. In a smaller single institution study, proton-pump inhibitor use, segment length greater than 3 cm and history of esophageal candidiasis were the key predictors beyond demographics, but information on smoking and use of NSAIDs or statins was not available [57]. Finally, an analysis involving 103 EAC cases from the U.K. General Practice Research Database found overweight and statins as a significant predictors, but not smoking [18].

The present calculator has limitations. While confounding factors were adjusted for in the underlying studies, the possibility of interactions among the factors has not been adequately examined. Thus, the calculations assume the risk factors act multiplicatively. This is a particular concern at more extreme risk estimates, where supporting data may be quite sparse. Although survival from EAC is poor (about 20% at five years), not all diagnosed with the cancer will die from it. This should be kept in mind when comparing estimated incidence rates of EAC in the thermometer with comparison rates which are mortality based. In addition, while mortality rates are specific for persons in the same age, sex and race group, within this group they represent population averages as they are not adjusted for other risk factors. Thus, risk of death from lung cancer or heart disease, for example, would be higher than shown for a smoker and lower than shown for a non-smoker. Use of a separate disease-specific risk calculator with additional disease-specific risk factors would be needed for more precise estimates. The less abundant and reliable data on progression to EAC in persons with BE should be considered in interpreting the absolute risk estimates in this setting. Since the BEACON studies used in the Fig. 2 analyses also contributed between 32 and 44% of cases to meta-analyses regarding four risk factors, the discriminative metrics may have some level of optimism. The relatively low ppv indicates that identification of additional predictive biomarkers, likely blood- and cytology-based in the general population, and biopsy-based among those diagnosed with BE, is greatly needed to improve discrimination accuracy in both groups [12]. Finally, it is applicable only to the US population.

The accuracy of all risk calculators depends upon the quality of the underlying data and generalizability to the targeted population. We drew upon multiple studies of various designs in arriving at a set of RR estimates. For some factors, such as smoking and BMI in the general population, this is straightforward given the published meta-analyses. For other factors, especially in the setting of BE, this was a more subjective process with ample opportunity for disagreement among experts. We addressed this by designing an open-source self-documenting application, with input parameters contained in few simple spreadsheets, which can be downloaded and modified to meet the needs of an institution or clinician. As a corollary, as technology, clinical practice and epidemiologic knowledge evolve, IC-RISC™ can be improved and kept relevant. For example, predictors based on germline mutations can be added as panels become larger, more predictive and widely available [58]. As non-endoscopic sampling methods are introduced into clinical practice (e.g. Cytosponge, encapsulated balloon), together with tailored assays incorporating somatic genetic and epigenetic abnormalities, they can also be included. [58–60] Its generic architecture also lends itself to being extended to other cancers with distinct pathways and/or intermediate stages, such as hepatocellular cancer, with discrete causes and rapidly developing screening and treatment modalities [61].

## Conclusions

Decisions regarding EAC prevention faced by patients and providers are plentiful. For example, they might involve preventative actions centered on lifestyle (e.g., weight loss, exercise, dietary change), chemopreventative actions (e.g., aspirin), non-invasive screening, endoscopic screening, surveillance, and treatment of high-risk Barrett’s. Informed judgment plays an important role in most of these decisions since compelling evidence does not currently exist to support hard decision thresholds. If decision thresholds did exist, they would have to account for vastly different ratios of risk to likely benefit for each option, and large inter-individual differences (in the general population as well as providers) in risk tolerance and willingness to undergo medical interventions, among many other factors. In this context, IC-RISC™ provides a simple and rapid means to graphically communicate risk of EAC in the context of other health risks, allows “what-if” scenarios regarding potential preventative actions, and can directly inform discussions regarding screening, surveillance and treatment options. It extends current qualitative clinical practice guidelines for endoscopic screening into a quantitative assessment, which brings the possibility of screening being offered to higher-risk persons not currently targeted (e.g., males without sGERD and females), while also potentially identifying lower-risk persons who might otherwise be targeted for screening as unlikely to benefit. It also may be useful in defining higher-risk persons for intervention trials. The calculator would benefit from prospective testing and application to existing well-characterized cohort studies to refine the estimates and facilitate harmonizing existing risk calculators so that a uniform message can be developed for clinical use. Until such time, IC-RISC™ can be used to inform joint decision-making rather than to indicate specific preventative actions or thresholds.

## Availability and requirements

Project name: IC-RISC.

Project home page: Software - https://github.com/FredHutch/IC-RISC-Working

Application – https://ic-risc.esocan.org

Operating system: Platform independent.

Programming language: R/Shiny.

License: Academic use - 2-Clause BSD; Non-academic - License needed.

## Additional file

Additional file 1: Supplementary methods, tables, figures and references. (DOCX 523 kb)

## Data Availability

The input datasets to the risk calculator are available at https://github.com/FredHutch/IC-RISC-Working (see “input_data”). Individual data from the selected BEACON studies used in the example cannot be made openly available due to ethical concerns (presence of four indirect identifiers.) Further information about the data and conditions for access are available from the corresponding author.

## Abbreviations

ACG: American College of Gastroenterology
BE: Barrett’s esophagus
BEACON: Barrett’s and Esophageal Adenocarcinoma Consortium
BMI: Body mass index
CI: Confidence interval
EAC: Esophageal adenocarcinoma
HGD: High-grade dysplasia
IC-RISC: Interactive and Contextual Risk Calculator
NSAIDs: Non-steroidal anti-inflammatory drugs
ppv: Positive predictive value
RR: Relative risk
sGERD: symptomatic gastroesophageal reflux disease
