# Supplementary material for: Interactive decision support for esophageal adenocarcinoma screening and surveillance
Source: BMC Gastroenterol. 2019 Jun 27;19:109. doi: 10.1186/s12876-019-1022-0 (PMC6598240; doi:10.1186/s12876-019-1022-0)
Supplement: Supplementary file 1 — Supplementary methods, tables, figures and references. (DOCX 523 kb) [file 12876_2019_1022_MOESM1_ESM.docx]

## Supplementary Methods

Risk Factors

Regarding risk factors for EAC in the general population (Supplementary Table 1), the number of cases included in the meta-analyses were: sGERD (n = 1,128)^1^, BMI (n = 1,197)^2^, smoking (n = 1,540)^3^, statins (n = 907)^4^, and NSAIDs (n = 1,226).^5^ The associations regarding, BMI and central obesity measures, and sGERD and smoking have been supported in cohort studies as well.^6,7^ Concerning NSAIDs, strong supporting evidence of a protective effect comes from a recent randomized controlled trial of aspirin plus/minus esomeprazole,^8^ and a meta-analysis of eight randomized clinical trials of aspirin.^9^ Physical activity associations were based on an analysis of pooled individual assessments of quantitative physical activity from 12 cohort studies of the National Cancer Institute Cohort Consortium involving 1.44 million participants; five of the studies contributed to analyses of EAC (n = 899).^10^ A linear model fit the data well (p non-linear = 0.97), so the reported hazard ratio (0.58, p trend = 0.01) for the 90^th^ percentile vs 10^th^ percentile levels of activity was used to calculate hazard ratios for quartiles. Supporting evidence for a slightly weaker inverse association with physical activity comes from a meta-analysis of published relative risks from case-control and cohort studies.^11^ The relative risk associated with family history was based on pedigrees from over 1,400 persons.^12^ The incidence of EAC among those who have been screened negative for BE is not well-studied since they are generally not followed clinically; a relative risk of 0.33 was estimated from a global model of EAC incidence in the general US population.^13^

Among persons with BE, many studies have estimated risk of EAC (and/or HGD) among persons with low-grade dysplasia with widely varying results (Supplementary Table 2.)^14–20^ For purposes of the risk calculator, we used the estimate from a recent large cohort study which, in addition to confirmed low-grade dysplasia, evaluated potential confounding by 10 risk factors in 2,697 BE patients followed for a median of 5.9 years (n = 154 EAC and/or HGDs.)^18^ Relative risks for persons with HGD or a DNA content abnormality were estimated from a meta-analysis and two prospective studies.^14,19,21^ BE segment length has been consistently linked with increased risk of EAC, although with considerable variation;^18,22,23^ again, the estimate by Parasa et al.,^18^ was chosen as representative. It is challenging to arrive at a sensible estimate for the association between sGERD frequency or severity and progression risk in persons with diagnosed BE, since i) most BE patients are diagnosed because of GERD symptoms, ii) those with symptoms are typically treated early in their clinical course with acid-reducing medications, and iii) few studies of BE patients have reported on associations between pre-medication sGERD and progression risk. However, it seems likely that severity of underlying reflux remains an important determinant of progression risk. First, recent studies indicate that use of acid-reducing medications (primarily proton pump inhibitors) reduce risk of progression in BE in a dose-response manner.^8,24^ Second, while sGERD is strongly related to EAC in the general population, with odds ratios approaching eight-fold in those with most frequent symptoms,^1^ community-based studies of BE prevalence report only a two- to three-fold increase in BE risk with sGERD, leaving an unexplained gap which is likely explained in BE progression.^25,26^ Under the assumption that the overall association of reflux with EAC is approximately evenly distributed between the two steps (development of BE and progression of BE), we used one half of the trend coefficient for the general population.

Regarding other potential risk factors, while a strong body of epidemiologic evidence suggests that infection with *Helicobacter pylori* is associated with an approximately 50% decreased risk of EAC,^27^ information on *H. pylori* status is not typically available in the primary care setting and was not included in the risk calculator. Similarly, there is some evidence from observational studies that diet, in particular higher intake of fat and lower intake of fruits and vegetables, is associated with increased EAC risk;^28–30^ however, a simple and accurate assessment of diet also is beyond the scope of the current calculator. While almost two dozen genetic loci have been identified as associated with EAC risk,^31^ their added discriminatory ability is rather modest and thus have not been included in the current version of the application.^32,33^

## Supplementary Figures and Tables


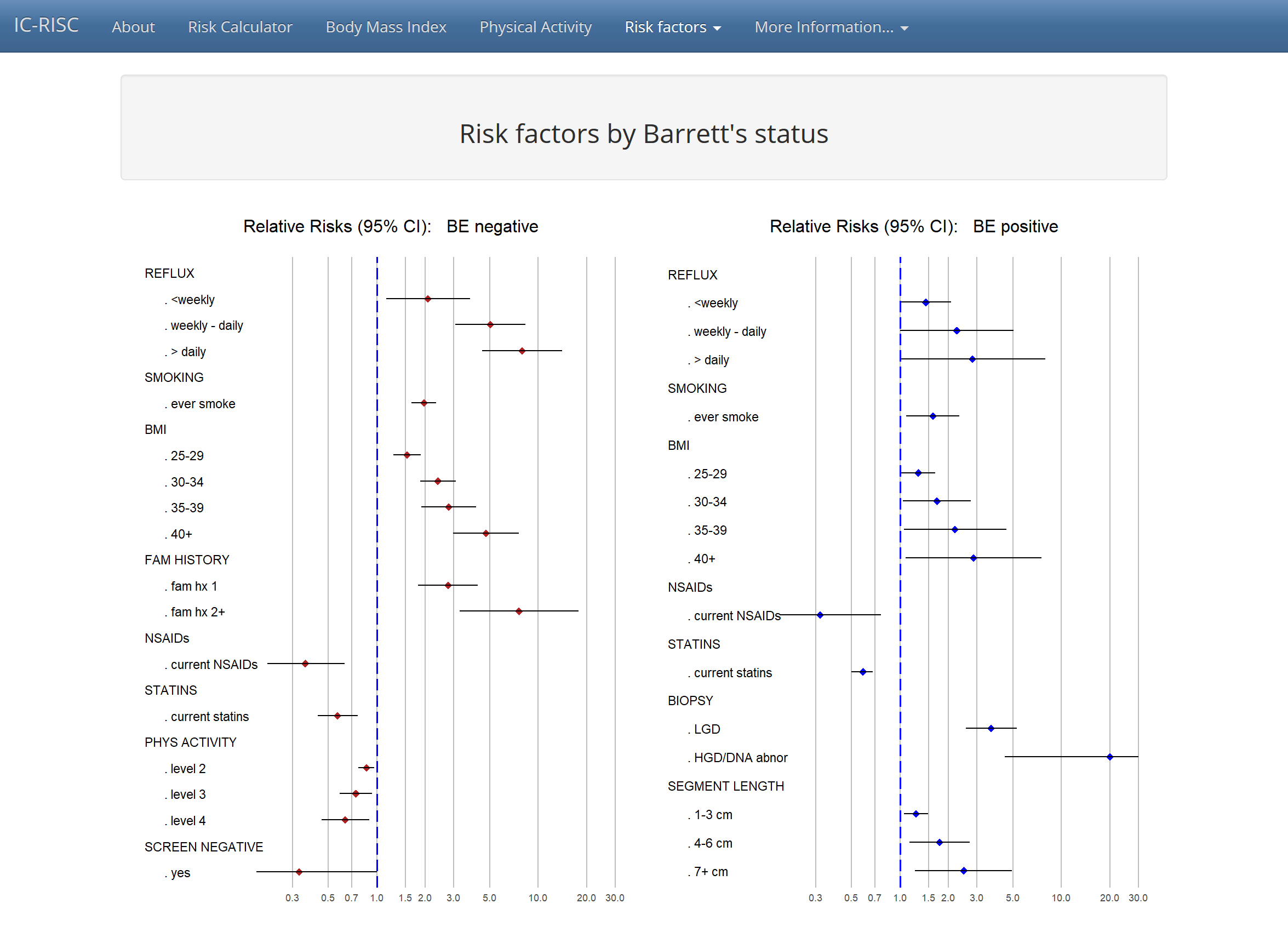


Supplementary Figure 1. Relative risk estimates and 95% confidence intervals (95% CI) for each predictor, stratified by Barrett’s esophagus status (unknown/negative vs. positive.)


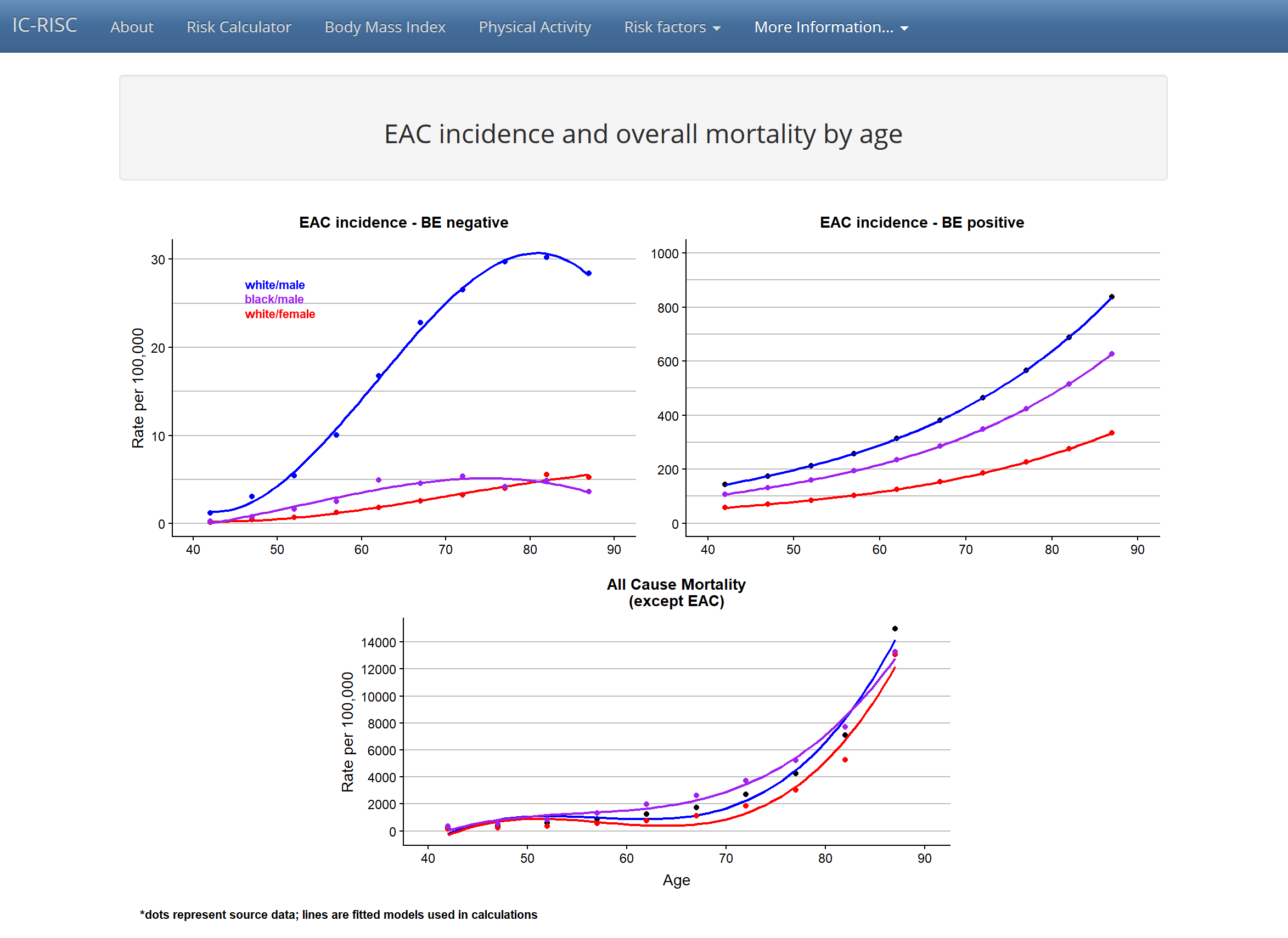


Supplementary Figure 2. Age-specific incidence and mortality rates (per 100,000) by sex and race for EAC stratified by Barrett’s esophagus status (unknown/negative vs. positive), and for all-cause mortality (excluding EAC).


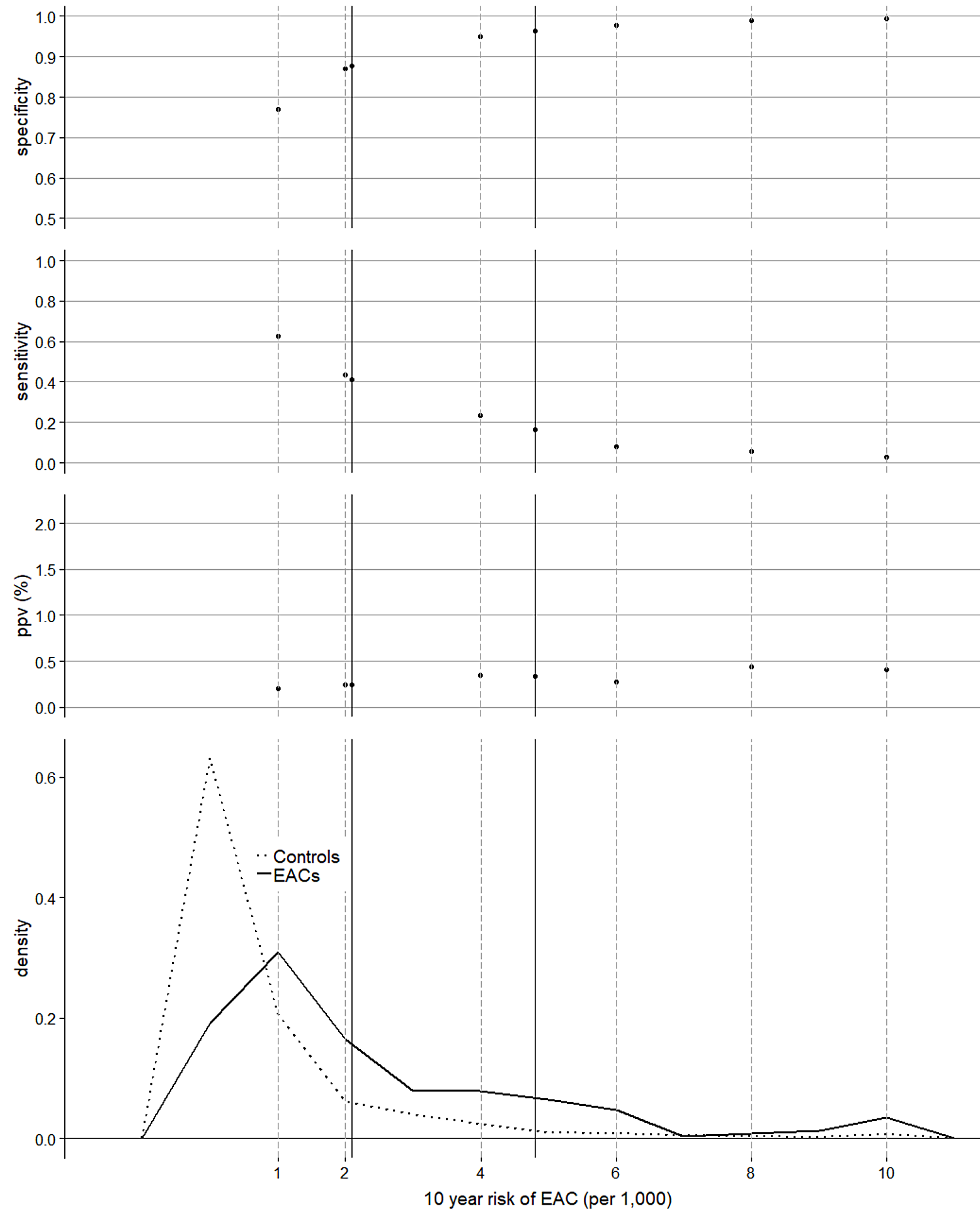


Supplementary Figure 3. Metrics describing estimated 10-year risk in EAC cases and controls **who have infrequent reflux** (less than weekly or rarely.) The bottom panel shows the distribution of 10-year risk estimates by case status. The two solid vertical lines represent examples of individuals for whom current ACG guidelines do not suggest that screening endoscopy be considered. The left-most (estimated risk of 2.1 per 1,000) corresponds to a 65-year-old ex-smoking black male with occasional reflux symptoms (“< weekly”), no family history, and a BMI of 36 (obese category II.) The right-most (4.8 per 1,000) corresponds to a 60-year-old non-smoking white male without reflux symptoms (“rarely”), a BMI of 31 (obese category I) and one first degree relative with BE/EAC. The top, second and third panels show how specificity, sensitivity and positive predictive value (ppv), respectively, vary according to possible thresholds for further action.

Supplementary Table 1. Estimates for relative risks (RR), 95% confidence intervals (CI), and prevalence in persons with unknown or negative Barrett’s esophagus status.

| **Risk Factor** | **RR** | **Lower CI** | **Upper CI** | **Prevalence** | **References/Notes** |
| --- | --- | --- | --- | --- | --- |
| REFLUX |  |  |  |  | **Cook MB, et al. *PLoS ONE* 9, e103508 (2014)**^1^ |
| rarely/never | reference |  |  | 0.51 |  |
| <weekly | 2.08 | 1.14 | 3.78 | 0.36 |  |
| weekly - daily | 5.07 | 3.07 | 8.37 | 0.09 |  |
| > daily | 7.96 | 4.51 | 14.05 | 0.04 |  |
| SMOKING |  |  |  |  | **Cook MB, et al. JNCI 102, 1344-53 (2010)**^3^ |
| never | reference |  |  | 0.41 |  |
| ever | 1.96 | 1.64 | 2.34 | 0.59 |  |
| BMI (kg/m^2^) |  |  |  |  | **Hoyo C, et al. Int J Epidem 41, 1706-18 (2012)**^2^ |
| <25 | reference |  |  | 0.45 |  |
| 25-29 | 1.54 | 1.27 | 1.87 | 0.41 |  |
| 30-34 | 2.39 | 1.85 | 3.08 | 0.11 |  |
| 35-39 | 2.79 | 1.89 | 4.13 | 0.03 |  |
| 40+ | 4.76 | 2.97 | 7.62 | 0.01 |  |
| FAMILY HISTORY |  |  |  |  | Sun X, et al. Cancer Epidem Bio Prev 25, 727-735 (2016)^12^ Chak A, et al. Cancer Epidemiol Biomarkers Prev 15(9):1668–73 (2006)^34^ |
| negative | reference |  |  | 0.93 |  |
| one | 2.76 | 1.81 | 4.21 | 0.07 |  |
| two+ | 7.62 | 3.27 | 17.76 | 0.001 |  |
| NSAIDs |  |  |  |  | **Rothwell PM, et al. Lancet 2011;377:31–41 (2011)**^9^ **Liao LM, et al. Gastro 142, 442-452 (2012)**^5^ |
| no | reference |  |  | 0.50 |  |
| current use | 0.36 | 0.21 | 0.63 | 0.50 |  |
| STATINS |  |  |  |  | **Thomas T, et al. J Gastrointest Cancer (2017)**^4^  Anon. Statistical Brief #459, https://meps.ahrq.gov/data_files/publications/st459/stat459.shtml^35^ |
| no | reference |  |  | 0.65 |  |
| current use | 0.57 | 0.43 | 0.76 | 0.35 |  |
| PHYS ACTIVITY |  |  |  |  | **Moore SC, et al. JAMA Intern Med 176, 816-825 (2016)**^10^ |
| quartile 1 | reference |  |  | 0.25 |  |
| quartile 2 | 0.86 | 0.77 | 0.96 | 0.25 |  |
| quartile 3 | 0.74 | 0.59 | 0.93 | 0.25 |  |
| quartile 4 | 0.64 | 0.45 | 0.90 | 0.25 |  |
| SCREEN NEGATIVE |  |  |  |  | Vaughan, TL & Fitzgerald, RC. Nat Rev Gastro Hepatol 12, 243–248 (2015)^13^ |
| no | reference |  |  | 0.98 |  |
| yes | 0.33 | 0.11 | 1.00 | 0.02 |  |

*Meta-analyses are in bold type.*

Supplementary Table 2. Estimates for relative risks (RR), 95% confidence intervals (CI), and prevalence in persons with diagnosed Barrett’s esophagus.

| **Risk Factor** | **RR** | **Lower CI** | **Upper CI** | **Prevalence** | **References/Notes** |
| --- | --- | --- | --- | --- | --- |
| REFLUX |  |  |  |  | RR based on one half of trend coefficient in general population, and an assigned p-value=0.05. Cook MB, et al. PLoS ONE 9, e103508 (2014)^1^ |
| rarely/never | reference |  |  | 0.1 |  |
| <weekly | 1.44 | 1.00 | 2.08 | 0.1 |  |
| weekly - daily | 2.25 | 1.00 | 5.08 | 0.4 |  |
| > daily | 2.82 | 1.00 | 7.97 | 0.4 |  |
| SMOKING |  |  |  |  | Hardikar S, et al. PloS one 8, e52192 (2013)^36^ Tan MC, et al. Aliment Pharm & Ther, http://onlinelibrary.wiley.com/doi/abs/10.1111/apt.14895^24^ |
| never | reference |  |  | 0.36 |  |
| ever | 1.60 | 1.10 | 2.34 | 0.64 |  |
| BMI (kg/m^2^) |  |  |  |  | RR based on estimated coefficient for grouped linear model with p(trend)=0.034. Krishnamoorthi R, et al. Gastro Endo 84, 40–46.e7 (2016).^37^ |
| <25 |  |  |  | 0.30 |  |
| 25-29 | 1.30 | 1.02 | 1.66 | 0.30 |  |
| 30-34 | 1.69 | 1.04 | 2.75 | 0.30 |  |
| 35-39 | 2.20 | 1.06 | 4.55 | 0.05 |  |
| 40+ | 2.86 | 1.08 | 7.55 | 0.05 |  |
| NSAIDs |  |  |  |  | Vaughan T, et al. Lancet Oncology 6:945–52 (2005)^38^ Jankowski JAZ, et al. Lancet 392, 400–408 (2018)^8^ |
| no | reference |  |  | 0.60 |  |
| current use | 0.32 | 0.14 | 0.76 | 0.40 |  |
| STATINS |  |  |  |  | **Thomas T, et al. J Gastrointest Cancer (2017)**^4^ Krishnamoorthi R, et al. Gastro Endo 84, 40–46.e7 (2016)^37^ |
| no | reference |  |  | 0.72 |  |
| current use | 0.59 | 0.50 | 0.68 | 0.28 |  |
| BIOPSY |  |  |  |  | Parasa S, et al. Gastro (2018)^18^ Krishnamoorthi R, et al. Am. J. Gastroenterol (2017)^17^ Duits et al. Gastro (2017)^16^  Rastogi T, et al. Gastrointestinal endoscopy (2008)^14^ Choi WD, et al. Gut (2017)^19^ Galipeau PC, et al. PLoS medicine 4 (2007)^21^ Anaparthy R, Sharma P. Nature Rev Gastro Hepatology (2014)^15^ Singh S, et al. Gastrointestinal Endoscopy (2014)^20^ |
| no abnormalities | reference |  |  | 0.86 |  |
| LGD | 3.68 | 2.56 | 5.31 | 0.11 |  |
| HGD/DNA abnor | 20.00 | 4.48 | 89.38 | 0.03 |  |
| SEGMENT LENGTH |  |  |  |  | Parasa S. et al. Gastroenterology 154, 1282-1289.e2 (2018)^18^ Sikkema M, et al. Am J Gastro 106, 1231-1238 (2011)^22^ Anaparthy R, et al. Clin Gastro Hepatol 11, 1430-1436 (2013)^23^ |
| <1 cm | reference |  |  | 0.40 |  |
| 1-3 cm | 1.25 | 1.06 | 1.49 | 0.30 |  |
| 4-6 cm | 1.76 | 1.15 | 2.71 | 0.20 |  |
| 7+ cm | 2.48 | 1.24 | 4.94 | 0.10 |  |

*Meta-analyses are in bold type.*

## Supplementary References

1. Cook MB, Corley DA, Murray LJ, et al. Gastroesophageal Reflux in Relation to Adenocarcinomas of the Esophagus: A Pooled Analysis from the Barrett’s and Esophageal Adenocarcinoma Consortium (BEACON). PLoS ONE 2014;9:e103508.

2. Hoyo C, Cook MB, Kamangar F, et al. Body mass index in relation to oesophageal and oesophagogastric junction adenocarcinomas: a pooled analysis from the International BEACON Consortium. Int J Epidemiol 2012;41:1706–18.

3. Cook MB, Kamangar F, Whiteman DC, et al. Cigarette smoking and adenocarcinomas of the esophagus and esophagogastric junction: a pooled analysis from the international BEACON consortium. J Natl Cancer Inst 2010;102:1344–53.

4. Thomas T, Loke Y, Beales ILP. Systematic Review and Meta-analysis: Use of Statins Is Associated with a Reduced Incidence of Oesophageal Adenocarcinoma. J Gastrointest Cancer 2017.

5. Liao LM, Vaughan TL, Corley D a, et al. Nonsteroidal anti-inflammatory drug use reduces risk of adenocarcinomas of the esophagus and esophagogastric junction in a pooled analysis. Gastroenterology 2012;142:442–452.e5; quiz e22–3.

6. Bodelon C, Anderson GL, Rossing MA, et al. Hormonal factors and risks of esophageal squamous cell carcinoma and adenocarcinoma in postmenopausal women. Cancer Prev Res Phila Pa 2011;4:840–50.

7. MacInnis RJ, English DR, Hopper JL, et al. Body size and composition and the risk of gastric and oesophageal adenocarcinoma. Int J Cancer 2006;118:2628–2631.

8. Jankowski JAZ, Caestecker J de, Love SB, et al. Esomeprazole and aspirin in Barrett’s oesophagus (AspECT): a randomised factorial trial. Lancet Lond Engl 2018;392:400–408.

9. Rothwell PM, Fowkes FGR, Belch JFF, et al. Effect of daily aspirin on long-term risk of death due to cancer: analysis of individual patient data from randomised trials. Lancet 2011;377:31–41.

10. Moore SC, Lee I, Weiderpass E, et al. Association of leisure-time physical activity with risk of 26 types of cancer in 1.44 million adults. JAMA Intern Med 2016;176:816–825.

11. Behrens G, Jochem C, Keimling M, et al. The association between physical activity and gastroesophageal cancer: systematic review and meta-analysis. Eur J Epidemiol 2014;29:151–170.

12. Sun X, Elston RC, Barnholtz-Sloan JS, et al. Predicting Barrett’s Esophagus in Families: An Esophagus Translational Research Network (BETRNet) Model Fitting Clinical Data to a Familial Paradigm. Cancer Epidemiol Biomarkers Prev 2016;25:727–735.

13. Vaughan TL, Fitzgerald RC. Precision prevention of oesophageal adenocarcinoma. Nat Rev Gastroenterol Hepatol 2015;12:243–248.

14. Rastogi A, Puli S, El-Serag HB, et al. Incidence of esophageal adenocarcinoma in patients with Barrett’s esophagus and high-grade dysplasia: a meta-analysis. Gastrointest Endosc 2008;67:394–8.

15. Anaparthy R, Sharma P. Progression of Barrett oesophagus: role of endoscopic and histological predictors. Nat Rev Gastroenterol Hepatol 2014;11:525–534.

16. Duits LC, Wel MJ van der, Cotton CC, et al. Patients With Barrett’s Esophagus and Confirmed Persistent Low-Grade Dysplasia Are at Increased Risk for Progression to Neoplasia. Gastroenterology 2017;152:993-1001.e1.

17. Krishnamoorthi R, Lewis JT, Krishna M, et al. Predictors of Progression in Barrett’s Esophagus with Low-Grade Dysplasia: Results from a Multicenter Prospective BE Registry. Am J Gastroenterol 2017;112:867–873.

18. Parasa S, Vennalaganti S, Gaddam S, et al. Development and Validation of a Model to Determine Risk of Progression of Barrett’s Esophagus to Neoplasia. Gastroenterology 2018;154:1282-1289.e2.

19. Choi W-T, Tsai J-H, Rabinovitch PS, et al. Diagnosis and risk stratification of Barrett’s dysplasia by flow cytometric DNA analysis of paraffin-embedded tissue. Gut 2017:gutjnl-2017-313815.

20. Singh S, Manickam P, Amin AV, et al. Incidence of esophageal adenocarcinoma in Barrett’s esophagus with low-grade dysplasia: a systematic review and meta-analysis. Gastrointest Endosc 2014;79:897-909.e4.

21. Galipeau PC, Li X, Blount PL, et al. NSAIDs modulate CDKN2A, TP53, and DNA content risk for progression to esophageal adenocarcinoma. PLoS Med 2007;4:e67.

22. Sikkema M, Looman CWN, Steyerberg EW, et al. Predictors for neoplastic progression in patients with Barrett’s Esophagus: a prospective cohort study. Am J Gastroenterol 2011;106:1231–1238.

23. Anaparthy R, Gaddam S, Kanakadandi V, et al. Association Between Length of Barrett’s Esophagus and Risk of High-grade Dysplasia or Adenocarcinoma in Patients Without Dysplasia. Clin Gastroenterol Hepatol 2013;11:1430–1436.

24. Tan MC, El‐Serag HB, Yu X, et al. Acid suppression medications reduce risk of oesophageal adenocarcinoma in Barrett’s oesophagus: a nested case-control study in US male veterans. Aliment Pharmacol Ther 2018;00:1–9.

25. Ronkainen J, Aro P, Storskrubb T, et al. Prevalence of Barrett’s esophagus in the general population: an endoscopic study. Gastroenterology 2005;129:1825–31.

26. Zagari RM, Fuccio L, Wallander MA, et al. Gastro-oesophageal reflux symptoms, oesophagitis and Barrett’s oesophagus in the general population: the Loiano-Monghidoro study. Gut 2008;57:1354–9.

27. Islami F, Kamangar F. Helicobacter pylori and esophageal cancer risk: a meta-analysis. Cancer Prev Res (Phila Pa) 2008;1:329–338.

28. Abnet CC, Corley DA, Freedman ND, et al. Diet and Upper Gastrointestinal Malignancies. Gastroenterology 2015;148:1234-1243.e4.

29. Silvera SAN, Mayne ST, Risch H, et al. Food group intake and risk of subtypes of esophageal and gastric cancer. Int J CancerJournal Int Cancer 2008;123:852–860.

30. O’Doherty MG, Cantwell MM, Murray LJ, et al. Dietary fat and meat intakes and risk of reflux esophagitis, Barrett’s esophagus and esophageal adenocarcinoma. Int J Cancer 2011;129:1493–1502.

31. Contino G, Vaughan TL, Whiteman D, et al. The evolving genomic landscape of Barrett’s esophagus and esophageal adenocarcinoma. Gastroenterology 2017.

32. Dong J, Buas MF, Gharahkhani P, et al. Determining Risk of Barrett’s Esophagus and Esophageal Adenocarcinoma Based on Epidemiologic Factors and Genetic Variants. Gastroenterology 2018;154:1273-1281.e3.

33. Kunzmann AT, Canadas Garre M, Thrift AP, et al. Information on Genetic Variants Does Not Increase Identification of Individuals at Risk of Esophageal Adenocarcinoma Compared to Clinical Risk Factors. Gastroenterology 2018.

34. Chak A, Ochs-Balcom H, Falk G, et al. Familiality in Barrett’s esophagus, adenocarcinoma of the esophagus, and adenocarcinoma of the gastroesophageal junction. Cancer Epidemiol Biomark Prev Publ Am Assoc Cancer Res Cosponsored Am Soc Prev Oncol 2006;15:1668–1673.

35. Anon. STATISTICAL BRIEF #459: Changes in Statin Therapy among Adults (Age ≥ 18) by Selected Characteristics, United States, 2000–2001 to 2010–2011. Available at: https://meps.ahrq.gov/data_files/publications/st459/stat459.shtml [Accessed September 22, 2018].

36. Hardikar S, Onstad L, Blount PL, et al. The role of tobacco, alcohol, and obesity in neoplastic progression to esophageal adenocarcinoma: a prospective study of Barrett’s esophagus. PloS One 2013;8:e52192.

37. Krishnamoorthi R, Borah B, Heien H, et al. Rates and predictors of progression to esophageal carcinoma in a large population-based Barrett’s esophagus cohort. Gastrointest Endosc 2016;84:40-46.e7.

38. Vaughan T, Dong L, Blount P, et al. Non-steroidal anti-inflammatory drugs and risk of neoplastic progression in Barrett’s oesophagus: a prospective study. Lancet Oncol 2005;6:945–52.
